# Treatment Effect of a Vascular-Disrupting Agent Dynamically Monitored by DWI: An Animal Experimental Study

**DOI:** 10.1155/2021/2909189

**Published:** 2021-12-30

**Authors:** Danping Huang, Ruimeng Yang, Yong Zou, Hongmei Lin, Xiangdong Xu, Xinhua Wei, Hanzheng Chang, Liqiong Wu, Wenshuang Ding, Wenjie Tang, Xinqing Jiang

**Affiliations:** ^1^Department of Radiology, The Second Affiliated Hospital, School of Medicine, South China University of Technology, Guangzhou, Guangdong 510180, China; ^2^Guangzhou Institute of Chemistry, Chinese Academy of Science, 510650 Guangzhou, China; ^3^Health Management Center, The Third Affiliated Hospital of Sun Yat-Sen University, Guangzhou, Guangdong 510630, China; ^4^Department of Pathology, The Second Affiliated Hospital, School of Medicine, South China University of Technology, Guangzhou, Guangdong 510180, China

## Abstract

**Objective:**

To investigate the treatment effect of a vascular-disrupting agent, M410, using diffusion-weighted imaging in a rabbit model of hepatic VX2 tumor.

**Methods:**

28 New Zealand white rabbit models with VX2 liver tumors were established and were randomly divided into M410 (intravenous injection of M410 at a dose of 25 mg/kg every three days) and control (intravenous injection of saline every three days) groups. Conventional and diffusion-weighted imaging (DWI) were acquired on a 3.0 T MR unit at baseline, 4 h, d 1, d 4, d 7, and d 14 posttreatment. B-value with 700 (s/mm^2^) was chosen during DWI examinations. Tumor volume and apparent diffusion coefficient (ADC) values of the entire tumor and solid component of the tumor at every time point were measured. Two randomly chosen rabbits from each group were sacrificed for H&E staining and CD34 immunohistochemical assessments at each time point. An independent sample *t*-test was used to assess differences in tumor sizes and ADC values of the entire tumor and solid component of tumors between two groups, with *P* < 0.05 considered statistically significant.

**Result:**

There was no significant difference in tumor volume between the two groups at baseline, 4 h, and d 1. With time, the tumors in the control group grew significantly faster than those in the M410 group, and the average ADC values of the M410 group were lower than those of the control group at d 1 and higher than those of the control group at d 4; as such, there were statistical differences between the two groups at these two time points but not at the other four time points. The following pathological results reflected the underlying morphological changes and vascular alterations.

**Conclusions:**

M410 performed well in inhibiting the growth of the hepatic VX2 tumor which could be noninvasively monitored by DWI metrics.

## 1. Introduction

Hepatocellular carcinoma is the second leading malignant tumor in the world. For years, sorafenib, a tyrosine kinase inhibitors (TKI) inhibitor, has been approved as a treatment option for patients with advanced HCC. Its activity is inhibition of the retrovirus-associated DNA sequences (RAS) protein /rapidly accelerated fibrosarcoma (RAF) protein/mitogen-activated and extracellular signal-regulated kinase (MEK)/extracellular signal-regulated kinase (ERK) signaling pathway. However, the efficacy of sorafenib is limited by the development of drug resistance. Since the diffusion of oxygen and nutrients in tissues remains limited, tumor angiogenesis is essential for the growth of tumors over 1–2 mm in diameter. The vascular system plays a key role in the survival and continuous growth of solid tumors, which are known to be significantly attributed to treatment failures. The tumor vasculature is structurally immature, with clutter and leaky phenotypes, and the inherent differences between tumor vessels and normal tissue vessels make tumor vasculature a unique target for the noninvasive treatment of malignant tumors.

Vascular disrupting agents (VDAs), unlike antiangiogenic therapies that inhibit the growth of new blood vessels, selectively target existing tumor vascular systems and disrupts the tumor vasculature [1–3]. Combretastatin A-4-phosphate (CA4P) is one of the most promising VDAs and has been widely used in targeted therapy and for the monitoring of tumors in various experimental studies [4–7]. It can disrupt the already formed vessel networks of growing tumors by inhibiting tubulin polymerization, resulting in extensive ischemic tumor necrosis. This selective effect can be attributed to the presence of actin as another type of cytoskeleton in normal endothelial cells but is lacking in tumor endothelial cells. Therefore, the normal vascular system is rarely affected by CA4P. The structural modification of molecules is known to be an effective way to obtain drugs with a better efficacy and lower toxicity, also providing an important way to develop new drugs.

In clinical practice, evaluating the therapeutic effect of VDAs on tumors often mainly depends on anatomical imaging, reflected by changes in the tumor's sizes. However, targeted therapies generally induce necrosis and cavitation rather than a significant tumor volume reduction, thereby limiting the capability of anatomic imaging to assess the efficacy of these new treatments [8]. This special effector mechanism of VDAs has led to the development of multiparameter imaging biomarkers to monitor early hemodynamic changes, cellular dysfunction, and metabolic disorders before tumor size changes can be detected, including computed tomography (CT), ultrasonography, dynamic contrast-enhanced magnetic resonance (MR) imaging, and diffusion-weighted (DW) MR imaging [8–11]. Among these imaging biomarkers, MR imaging is currently the most commonly used noninvasive method for evaluating the response of malignant tumors to treatments; a complete set of MRI-derived parameters has been used as morphological, physiological, and metabolic/molecular biomarkers for the detection and characterization of malignant tumors and evaluation of therapeutic treatment effects [4, 12–15]. DWI technology, which can reflect the essential changes in histology and cytology, performed well in the evaluation of early response monitoring of tumors after antivascular treatment with VDAs [16]. DWI has been used in animal tumor models to distinguish between living and necrotic tumor tissue. This MR imaging technique can describe molecular diffusion, i.e., the Brownian motion of water protons in biological tissues. The rate of the diffusion of microscopic water within tissues can be measured by using different gradient durations, amplitudes, and b-values; the calculated diffusion coefficient is termed apparent diffusion coefficient (ADC). The diffusion of microscopic water within tissues is also influenced by the cellular membranes and the extracellular matrix. A relatively high cell number will yield relatively low ADC values; therefore, DWI can be used to monitor early responses to antiangiogenic therapy, which is seen as changes in the ADC values of the tumor.

VDAs have entered clinical trials for over 16 years. CA4 analogue (Z)-3, 4′, 5-trimethyl-isopentene-3′-o-phosphate disodium (M410), which was used in the present study, is a new CA4P analogue which can inhibit tumor cell growth by restricting microtubulin polymerization, leading to the mitotic block of its intrinsic vascular endothelial cell [1, 2, 17]. Previous study [1] have shown that M410 was an effective inhibitor of bovine brain tubulin polymerization in vitro and inhibit the growth of human colon cancer xenografts in vivo in nude mice. Yang et al. [2] confirmed the mechanism of the inhibitory effects of M410 on a MDA-MB-231 breast cancer cell line. To the best of our knowledge, M410 is still rarely studied in human liver cancer. Therefore, DWI was employed in this study to dynamically monitor the tumor responses to the VDA M410 based on a rabbit model of hepatic VX2 tumor.

## 2. Materials and Methods

### 2.1. Experimental Animal Model

The institutional ethics committee approved this study for animal care and use. Twenty-eight male New Zealand white rabbits (weight, 2.0–2.5 kg) were used in this study, provided by the Center of Laboratory Animal Science of Guangdong Province. VX2 carcinomas were implanted into the left lobe of the livers of 28 experimental rabbits. The tumors were allowed to grow in the rabbit liver for about 2 weeks to a diameter exceeding 1 cm in order to effectively generate a rabbit VX2 implanted liver cancer model.

### 2.2. Grouping and Experimental Design

A total of 28 rabbit models with liver cancer were successfully established. These were randomly divided into M410 (*n* = 14) and control groups (*n* = 14). MRI scans were performed before (baseline) and 4 hour, 1 day, 4 days, 7 days, and 14 days (4 h, d 1, d 4, d 7, and d 14, respectively) after the administration of the VDA. After the baseline scan, the rabbits in the M410 group were intravenously injected via the marginal ear vein with M410 (a combretastatin derivative VDA provided by Guangzhou Institute of Chemistry, Chinese Academy of Sciences), which was dissolved in 2 ml of sterile saline at a dose of 20 mg/kg. An equal volume of sterile saline was injected intravenously in the control group. The treatment was performed once every three days (day 0, 3, 6, 9, and 12). Two randomly selected rabbits were sacrificed via a vascular air embolism after each scan time point. Tumor samples were then harvested and cut into slices (of equal thickness) paralleling the transverse scan plane after which they were immediately fixed in a neutral formaldehyde solution (containing 4% formaldehyde). Finally, the specimens were sent to pathologists for H&E and CD34 immunohistochemical staining.

### 2.3. Magnetic Resonance Imaging

After deep anesthetization with urethane (4 ml/kg), the rabbits were placed in a supine position. An MRI was performed on a 3.0-Tesla MR system (Verio; Siemens, Erlangen, Germany) with a soft coil. Scan parameters were as follows: axial and coronal fast spin-echo T2WI, axial T1WI (fast low angle and shot, FLASH), and DWI (spin-echo echo planar imaging, SE-EPI) with a section thickness of 3.0 mm, intersection gap of 0 mm, field of view of 150 × 150 mm, imaging acquisition matrix of 256 × 256, b-values of 700 s/mm^2^, and an acquisition time of about 30 min.

### 2.4. Tumor Volume Measurement

Tumor-containing slices were chosen on the basis of T2WI, and then a manual region of interest (ROI) delineation of the tumors was carried out. The total area of the tumor was calculated using the following formula:

(1) $$\begin{array}{c} \text{Tumor volume}=\sum \text{Tumor area on each tumor containing slice}\times \left(\text{Slice thickness}+\text{Gap}\right). \end{array}$$

### 2.5. ADC Value Measurement of the Entire and Solid Component of Tumor

Each tumor-containing slice was chosen based on ADC maps, then followed by the area of tumor delineation using an operator-defined ROI method. The ADC value of the entire tumor was calculated after summation and then divided by the number of slices to obtain the ADC of entire tumor according to

(2) $$\begin{array}{c} \text{ADC of entire tumor}=\sum \left(\frac{\text{ADC of each tumor containing slice}}{\text{number of slices}}\right). \end{array}$$

Furthermore, central tumor-containing slices were selected based on ADC images. Three circular ROIs containing at least 40 pixels were delineated in the solid component of tumor, and the mean value was calculated as the ADC value of the solid component of the tumor.

To assess the dynamic changes in the tumor after treatment with M410, the ADC values of the entire tumor and solid component of the tumor in the M410 group at two consecutive time points were compared. To compare the difference in the ADC change between the two groups at the same time point, ADC values were converted into ∆ADC% according to the following formula:

(3) $$\begin{array}{c} \Delta \text{ADC}\%=\frac{\left(\text{ADC of any time point}-\text{ADC of baseline}\right)}{\text{ADC of baseline}\times 100\%}. \end{array}$$

### 2.6. Pathological Examination

Two rabbits in each group were sacrificed immediately after the MR scan at every time point; the central tumor-containing slice was selected, and the tumor was cut in the axial plane corresponding to the plane of the MR images, after which the tumor slices were fixed in 10% formaldehyde solution, embedded in paraffin, and stained with H&E and CD34. Finally, the histological sections were studied visually and analyzed under a light microscope.

### 2.7. Statistical Analysis

Statistical analyses were performed using SPSS version 23.0 (SPSS, Chicago, IL, USA). Numeric data were reported as the mean ± standard deviation. The tumor volume and ADC values at each time point between the experimental and the control group were compared by two independent sample *t*-tests. A *p* value of less than 0.05 was considered to indicate a significantly statistical difference.

## 3. Results

### 3.1. MRI Findings at Baseline

**Figure 1:**
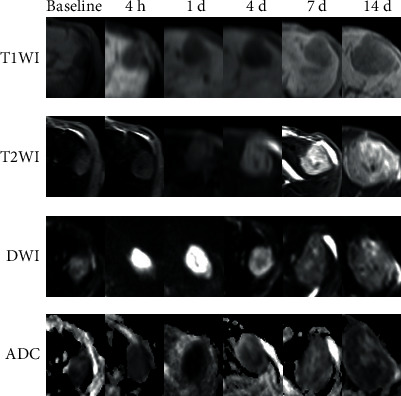
Findings in the M410 group. At all-time points, the tumor was hypointense on T1-weighted imaging (T1WI) and heterogeneously hyperintense on T2-weighted imaging (T2WI). At 4 h and 1 day after VDA treatment, the diffused weighted-imaging (DWI) signal of the tumor increased compared with the baseline, and the apparent diffusion coefficient (ADC) values of both the entire tumor and the solid component of the tumor decreased. At 4–14 days after treatment, the DWI signal of the tumor gradually decreased, and the ADC value of the entire tumor gradually increased compared with day 1. The ADC value of the solid component of the tumor first increased on day 4, then gradually decreased on day 7 and day 14 compared with that on day 1.

A total of 28 rabbit liver cancer models were successfully established. At baseline, the tumors had an oval shape with a clear border easily demarcated from the surrounding normal liver tissue in all imaging sequences. The tumor appeared hyperintense on T2WI and hypointense on T1WI images. In the center of several tumors, minute dots or irregular foci of necrosis were seen, which appeared more hyperintense and hypointense on T2WI and T1WI images, respectively. On ADC maps, the tumor appeared hypointense with a hyperintense center (Figure 1).

### 3.2. Tumor Volume

The mean tumor volume of the two groups was calculated on the basis of T2WI images at each time point. The mean tumor volume increased slowly between the two groups from baseline to d 1, increasing rapidly thereafter from d 4 to d 14, especially in the control group. There were statistically significant differences in the tumor volume between the two groups at the time points corresponding to d 4, d 7, and d 14 (M410 group vs. control group: 2 063 ± 1 541 vs. 3 350 ± 1 322 mm^3^, 3 325 ± 2 328 vs. 7 421 ± 3 177 mm^3^, and 7 518 ± 3 045 vs. 22 000 ± 2 653 mm^3^, respectively; *P* < 0.05), and no statistically significant differences were found at the other three time points (baseline, 1 076 ± 613 mm^3^ in the M410 group vs. 1 273 ± 334 mm^3^ in the control group; 4 h, 927 ± 472 vs. 1 108 ± 200 mm^3^; and d 1, 1 218 ± 939 vs. 1 308 ± 284 mm^3^, *P* < 0.05) (Table 1); thereby, suggesting that the tumor in the control group grew faster than that in the M410 group at the time points corresponding to d 4, d 7, and d 14, which indicates that M410 inhibited tumor growth.

### 3.3. Dynamic Changes in Mean ADC Values of the Entire Tumor and Solid Component of Tumor

**Figure 2:**
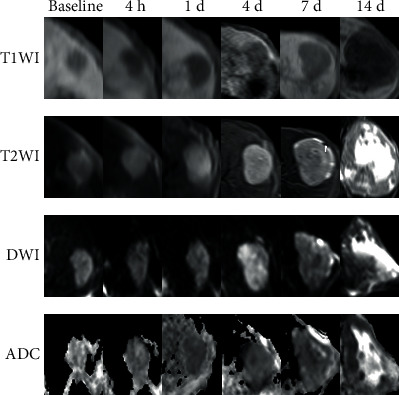
Findings in the control group. The tumor remained hypointense on T1-weighted imaging (T1WI) at all time points. Similarly, the tumor was hyperintense on T2-weighted imaging (T2WI) throughout the experiment, with obvious liquefaction necrosis in the center of the tumor at day 14. At baseline, the tumor was hyperintense on diffused weighted-imaging (DWI), from 4 h to day 14. The DWI signal of the tumor gradually increased compared with the baseline, and the apparent diffusion coefficient (ADC) value of the entire tumor decreased from the baseline till day 4, after which it increased continuously until day 14 due to the liquefaction necrosis in the center of the tumor. However, the ADC value of the solid component of the tumor decreased gradually throughout the experiment.

**Figure 3:**
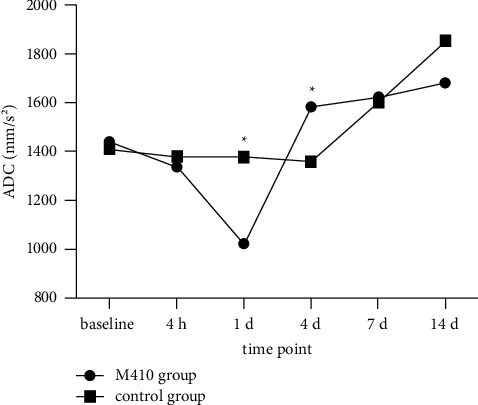
Changes in the mean apparent diffusion coefficient (ADC) value of the entire tumor of two groups at different time points. The mean ADC values of the entire tumor of the M410 group decreased from the baseline to day 1, then increased gradually until day 14. The control group values fell slightly from the baseline to day 4, then increased until day 14. There were statistically significant differences in the mean ADC values of the entire tumor between the two groups at the time points of d 1 and d 4 (*P*=0.032, 0.012).

**Figure 4:**
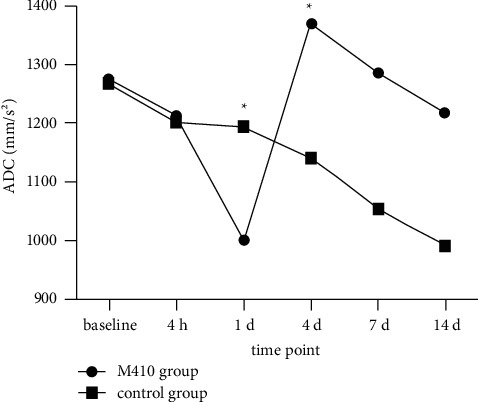
Changes in the mean apparent diffusion coefficient (ADC) from the solid component of the tumor of two groups at different time points. The mean ADC values of the M410 group of tumor parenchyma decreased from the baseline to d 1, then increased until d 4, and decreased again until d 14. The control group values fell continuously from the baseline to d 14. There was a statistically significant difference in the mean ADC values of the entire tumor between the two groups at the time points of d 1 and d 4 (*P*=0.04, *P*=0.045).

**Figure 5:**
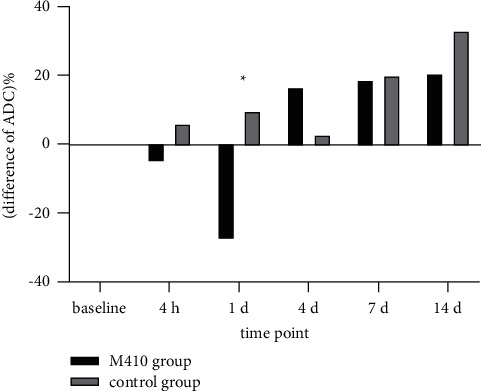
Comparison of the difference in the apparent diffusion coefficient (ΔADC%) of the tumor between the two groups at different time points. There was a decrease-increase course for ΔADC% in the M410 group and an increase-decrease-increase course in the control group, as shown by the images above. The ΔADC% in the M410 group was significantly lower on day 1 than that in the control group. There was a significant difference in the ΔADC% between the two groups. (^*∗*^*P*=0.046).

The mean ADC values of the entire tumor first in the M410 group decreased, then increased gradually; similarly, the control group values also fell slightly at first, after which they increased. There were statistically significant differences in the mean ADC values of the entire tumor between the two groups at the time points corresponding to d 1 and d 4; there was no statistically significant difference at the other four time points (Table 2). The mean ADC values of the solid component of tumor in the M410 group decreased at first, then increased, and decreased again. However, the control group values fell continuously. There were statistically significant differences in the mean ADC values of the solid component of the tumor between the two groups at the time points corresponding to d 1 and d 4, but there were no statistically significant differences at the other four time points (Table 2) (Figures 1–5).

### 3.4. Tumor Histological Dynamic Changes after M410 Administration

**Figure 6:**
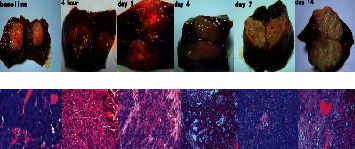
Gross pathology of the rabbit VX2 liver tumor model and hematoxylin and eosin staining (×400) of the tumor in the M410 group at baseline, 4 h, 1, 4, 7, and 14 days after treatment. The intrahepatic mass is clear without capsule formation, and the tumor is grayish white and similar to a fish flesh. There are multipoints hemorrhagic foci in the tumor. The tumor was tough and clearly demarcated with the surrounding liver tissue. Corresponding under the light microscope observation, from the baseline to day 14, revealed that the tumor cells were larger in size, irregular in morphology, disordered in arrangement, and the nuclear atypia was obvious. Scattered patchy or focal necrosis and partial fusion were observed in the tumor tissue. The new capillaries in the interstitium were more abundant, and the fibrous connective tissue grew into bundles.

**Figure 7:**
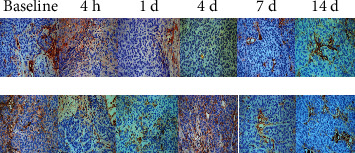
CD34 staining (×400) of the tumor at baseline, 4 h, 1, 4, 7, and 14 days after treatment in the two groups. From 4 hours and day 1 to day 4, a decrease in the tumor blood vessels and an increase on day 7 and 14 were observed in the M410 treated group (the first line). However, there was no such change in the control group; tumor blood vessels were abundant at all time points in the saline-treated group (the second line).

The tumors appeared oval in shape and grey in color in the macrograph, alike a fish meat sample (Figure 6), four hours and 1 day after administration of M410. The membranes of most of the cells were still intact under light microscopy, despite the heavy edema detected in tumors. Cell edema and the reduction of the extracellular space were responsible for the decrease in ADC values in the periphery as compared with those in the controls. On d 4, the tumor blood vessels were reduced, which was validated by a CD34 immunohistochemical stain, confirming the presence of focal necrosis and irregular foci of hemorrhage in the tumor tissue. The mean ADC values increased significantly in both central and peripheral regions compared with those on d 1, leading to an increase in the ADC value of the entire tumor and solid component of the tumor. On d 7 and d 14, liver sections showed signs of central necrosis mixed with regrowing tumor in the peripheral tumor; due to newborn blood capillaries, the mean ADC value increased gradually in the center, but the ADC value decreased in peripheral regions compared with those on d 4, which lead to the gradual increase in the ADC value of the entire tumor but resulted in a decrease in the ADC value of the solid component of tumor. The surrounding normal hepatic tissue did not show any significant change (Figures 6-7).

## 4. Discussion

In our study, we used DWI for the first time monitoring the changes of rabbit VX2 liver tumor after treatment with a novel combretastatin A4 analogue, M410, at a cellular level and then made a comparative analysis with the histopathological results. We found that, M410 had an obvious inhibitory effect on the growth of tumor; specifically, there was a reduction on the number of tumor cells and blood vessels during the period from baseline to d 4, but there were an obvious increase of tumor necrosis, viable cells at the periphery, and tumor blood vessels during the middle and later periods of the treatment (d 4–d 14).

VDAs selectively destroy tumor endothelial cells and inhibit microtubule polymerization, leading to the deformation and swelling of tumor vascular endothelial cells, followed by the coagulation and occlusion of tumor vessels, and finally leading to the secondary ischemic necrosis of tumor cells. M410 in this study is a newly synthesized combretastatin derivative with a better efficacy and lower toxicity than CA4P [1, 18]. In an in vitro experiment, Cai et al. found that M410 showed the strongest antiproliferative activity against tumor cells out of 6 newly synthesized compounds with the MTT method [1–3].

It is well known that the ADC value obtained from DWI reflects the cell structure, and that a relatively high cellularity will yield a relatively low ADC value. The signal intensity in the DWI does not only reflect the diffusion but also the T2 value of the tissue; the ADC map with a high b-value is used to combat this so-called T2 shine-through effect [19]. In our study, an image with a good signal-to-noise ratio was obtained with a b-value of 700 s/mm^2^.

Although VDAs can result in a selective vascular shutdown and tumor necrosis, these cannot directly result in tumor volume reduction. Changes in cellularity occur in the early stages of treatment, meaning that changes in the ADC value may precede changes in the tumor size. In this study, from baseline to 4 h and d 1, there were no significant changes in the tumor volume, but the ADC values gradually declined, especially on d 1; pathological findings confirmed that the tumor cells were ischemic, hypoxic, and swollen. However, the intercellular connections remained intact, thereby, restricting the diffusion of free water molecules into the interstitial space; until 4 days after treatment with M410, when cellular edema further increased, cellular membranes broke down, and a large number of water molecules entered the interstitial space. At this point, ADC values of both entire tumor and solid component of the tumor increased to values which exceeded those of the control group (*p* < 0.05), indicating that the inhibition of the tumor growth was obvious after treatment with M410; due to further necrosis, the ADC value of the entire tumor continued to increase on d 7 and d 14. Although histological findings revealed cytolysis in the center of the tumor, and viable tumor cells at the periphery became thicker, leading to an increase in the tumor volume, the ADC value of the solid component of the tumor decreased. Cai et al. found similar results when investigating the 12-day dynamic characteristics of tumor responses to the intravenous administration of CA4P in rabbit VX2 tumor models [18].

The regrowth along the rim of the tumor contribute to the increase in the tumor volume and the decrease in the ADC value. The nutrients and oxygen supporting this growth are partially obtained from neighboring normal host tissues. The change in the tumor phenotype after treatment may be an another contributing factor. Hypoxic conditions in the tumor after treatment with VDA may upregulate hypoxia-inducible factor 1*α* (HIF-1*α*), which stimulates the expression of angiogenic genes, increases the level of vascular endothelial growth factor (VEGF), and improves the process of angiogenesis. Therefore, VDAs are suggested to be combined with other chemotherapy drugs to further improve cancer treatment effects. Aboubakr et al. [20] studied the antitumor activities of CA4P and vincristine against liver cancer in rats and found that the anticancer activity of drugs with a narrow therapeutic window such as vincristine may be greatly improved by its coadministration with CA4P, thereby, enhancing its activity and causing fewer side effects [21].

The duration of inhibition of functional blood vessels may depend on the VDA dose and tumor model. In this study, we chose a repeated intravenous injection (every three days) scheme to study the interference effect of M410 on solid tumor blood vessels in vivo. We found that M410 can selectively close blood vessels in rabbit liver tumors at clinically relevant doses and that repeated intravenous injections can prolong vascular closure time [22]. CD34 staining showed that the tumors decreased at 4 h and recovered at d 7 after treatment, indicating that the inhibition effect of M410 on the rabbit VX2 liver tumor was prolonged by the repeated intravenous injection scheme. A rabbit VX2 liver tumor is the most convenient and commonly used model to simulate human primary or secondary liver cancer due to its similar blood supply, genotype, and metabolism with advanced human liver cancer [23, 24]. Other animal model for angiogenesis evaluation, such as CAM (chick embryo chorioallantoic membrane), due to the natural immune deficiency, the chick embryo may receive transplantations from different tissues and species without immune response. The chick CAM model is faster than mammalian models, where tumor growth needs 3 and 6 weeks, and the ACM model is also simple and low cost, which makes it better and more popular. The major disadvantage of the CAM model is that most tumor cells are unable to form visible colonies in secondary organs due to the short time (8–10 days) between implantation and chick hatching. Another disadvantage is that the CAM already contains a well-developed network of blood vessels, and new vessels is hardly indistinguishable from hemangiectasis and rearrangement of preexisting vessels [25].

The efficacy of VDAs also depends on factors such as tumor size, nitric oxide level, interstitial fluid pressure, and vascular permeability; however, these factors vary by the tumor type. We hypothesized that the tumor tissue would significantly increase in the control group at the later stage of the experiment due to the lack of an effect of M410, and the mean ADC values of the entire tumor would be lower than those of the M410 group. However, the mean ADC values of the entire tumor of the control group was higher than those of the M410 group 14 days after treatment with M410. This may be due to the ischemia and necrosis in VX2 tumors, which lead to an increase in the mean ADC values of the entire tumor in the control group at a later stage of the experiment and also concealed the decrease in the ADC value of the solid component of the tumor caused by tumor cell hyperplasia. Li et al. [26] found that the degree of necrosis in the rabbit VX2 tumor gradually increased since the occurrence of necrosis within the 4-week time span of their study, proving that VX2 tumors are prone to ischemia and necrosis.

In the field of drug therapy for malignant tumors, the tumor vascular system is one of the potential targets. The growth and metastasis of the solid tumor are dependent on the vascular system, which can provide essential oxygen and nutrients. By directly acting on the tumor vascular system, VDAs, therefore, have an important role in the prevention and inhibition of tumor growth. However, hypoxic conditions after VDA treatment for the tumor may upregulate HIF-1*α*, which further increase the level of VEGF. VEGF is not merely a key proangiogenic element but is also immune modulator, which boosts vascular formation and cooperates in creating permissive environment in most lethal malignancies and leads to poor drug response and survival [27]. That may explain the presence of an inevitable viable rim of tumor cells survived at the periphery after VDA treatment. Recently, the cancer immunotherapy has revolutionized the way we treat cancer patients in the past few years, and immune checkpoint inhibitors (ICIs) are currently the most successful immunotherapy approach. However, anti‐programmed death 1(PD‐1), anti‐programmed death ligand 1 (PD‐L1), and anti-cytotoxic T lymphocyte antigen 4 (CTLA‐4) monotherapy in clinical studies showed that ICIs still had limited efficacy. Combination therapy with different ICIs, chemotherapy, or antitumor angiogenic agents can significantly improve the efficacy, so, new combinations of VDAs with other chemotherapeutic agents and immunomodulators may have great potential to inhibit tumorigenesis and release effective immune responses.

There were some limitations to this study. First, the sample size was small since it is difficult and time-consuming to perform a study including a large number of rabbit VX2 models of primary liver cancer with continuous MR time points scanning, which also turned out to be a large scanning workload. From our results, 28 experimental rabbits employed in this study have generally fulfilled our hypothesis of using MR-DWI to quantitatively and dynamically monitor the therapeutic effect of the novel VDA (M410) on liver cancer. Second, the MR scan was performed on a clinical scanner with an otherwise animal MR coil. Third, the motion artifact of animal models, such as heartbeats and abdominal respiration, could not be completely eliminated during MRI examination. Fourth, only one treatment medicine, M410, was chosen instead of drug combination. Finally, animal tumor models are different from human beings, and the results cannot fully represent the histological characteristics of liver cancer in clinic, which we will figure out new animal models in the future studies.

In conclusion, M410 is an effective VDA which inhibits tumor growth by delaying the increase in the tumor volume compared with the control group. Moreover, DWI is a promising technology to noninvasively monitor the response of tumors to VDA therapies, and the proliferation or necrosis of rabbit VX2 tumors can be reflected in the dynamic changes in ADC and ADC% values of the tumor.

## Figures and Tables

**Table 1:** Average tumor volume of two groups at each time point (mm^3^).

Time	M410 group	Control group	*P* value
Baseline	1 076.00 ± 613.54	1 273.40 ± 334.48	0.300
4 h	927.42 ± 472.95	1 108.50 ± 200.40	0.235
d 1	1 218.40 ± 939.26	1 308.00 ± 284.69	0.131
d 4	2 063.13 ± 1 541.41	3 350.50 ± 1 321.54	0.046^*∗*^
d 7	3 325.50 ± 2 328.4	7 424.67 ± 3 177.35	0.025^*∗*^
d 14	7 518.75 ± 3 045.68	22 000.5 ± 2 653.94	0.021^*∗*^

^*∗*^ *P* value <0.05 is significant.

**Table 2:** The average ADC values of the entire tumor and the solid component of the tumor in two groups (mean ± SD) mm^2^/s.

Time	Entire tumor		*P* Value	Solid component of the tumor		*P* value
	M410 group	Control group		M410 group	Control group	
Baseline	1 440.52 ± 180.34	1 409.34 ± 330.79	0.759	1 275.88 ± 236.35	1 266.62 ± 290.70	0.927
4 h	1 338.39 ± 171.49	1 380.28 ± 95.27	0.467	1 212.66 ± 225.05	1 200.69 ± 204.76	0.888
d 1	1 020.61 ± 138.18	1 377.79 ± 467.27	0.032^*∗*^	1 000.45 ± 168.00	1 193.97 ± 219.06	0.04^∗^
d 4	1 581.98 ± 190.98	1 360.69 ± 103.18	0.012^*∗*^	1 369.76 ± 230.76	1 140.58 ± 183.17	0.045^∗^
d 7	1 621.26 ± 224.87	1 600.43 ± 220.75	0.875	1 284.89 ± 321.94	1 054.57 ± 209.61	0.173
d 14	1 680.36 ± 80.74	1 855.25 ± 158.95	0.097	1 218.34 ± 180.30	991.08 ± 163.56	0.111

ADC; apparent diffusion coefficient, ^*∗*^*P* value <0.05 is significant.

## Data Availability

All the data used to support the findings of this study are available from the corresponding author upon request.
